# Striving for a more person-centered psychosis care: results of a hospital-based multi-professional educational intervention

**DOI:** 10.1186/s12888-020-02871-y

**Published:** 2020-11-04

**Authors:** Katarina Allerby, Anneli Goulding, Lilas Ali, Margda Waern

**Affiliations:** 1 Section of Psychiatry and Neurochemistry, Institute of Neuroscience and Physiology, Sahlgrenska Academy, University of Gothenburg, Blå Stråket 15, 41345 Gothenburg, Sweden; 2 Psychosis Department, Region Västra Göraland; Sahlgrenska University Hospital, 41345 Gothenburg, Sweden; 3 Department of Psychology, University of Gothenburg, Haraldsgatan 1, 41314 Gothenburg, Sweden; 4 Institute of Health Care Sciences, Centre for Person-Centred Care, Sahlgrenska Academy, University of Gothenburg, Box 100, 40530 Gothenburg, Sweden; 5 Psychiatry Department, Region Västra Götaland, Sahlgrenska University Hospital, 41345 Gothenburg, Sweden

**Keywords:** Schizophrenia, Psychosis, Person-centered psychosis care, Person-centered care, Patient outcomes, Empowerment, Consumer satisfaction

## Abstract

**Background:**

Reluctance on the part of mental health professionals constitutes an important barrier to patient participation in care. In order to stimulate person-centeredness in the inpatient care of persons with psychotic illness, we developed and tested an educational intervention for hospital staff (including psychiatrists) at all four wards at the Psychosis Clinic, Sahlgrenska University Hospital in Gothenburg, Sweden. The intervention was co-created by professionals, patients, and researchers using a participatory approach. In addition to lectures and workshops, staff created and implemented small projects to increase person-centeredness on their own wards. A primary focus was to establish a partnership between patient and staff by capturing and utilizing the patient’s narrative to support active engagement in the care process. This included the development of a person-centered care plan. We hypothesized that the intervention would be associated with increased patient empowerment (primary outcome) and satisfaction with care (secondary outcome).

**Methods:**

A before and after design was used to test group differences in patient empowerment (Empowerment Scale) and consumer satisfaction (UKU-ConSat Rating Scale). All patients receiving inpatient psychosis care during measuring periods were eligible if meeting inclusion criteria of schizophrenia spectrum disorder, age > 18, and ability to comprehend study information. Severe cognitive deficit and inadequate Swedish language skills were exclusion criteria. Data on possible confounding variables including overall health (EQ-5D), symptom burden (PANSS), and functional ability (GAF) were collected alongside outcome measures.

**Results:**

ANCOVAs with overall health as a confounding variable showed no group differences regarding empowerment before (*n* = 50) versus after (*n* = 49) intervention, sample mean = 2.87/2.99, *p* = .142, *eta*^*2*^ = .02, CI = -.27–.04. Consumer satisfaction (*n* = 50/50) was higher in the post-implementation group (4.46 versus 11.71, *p* = .041 *eta*^*2*^ = .04, CI = -14.17– -.31).

**Conclusion:**

The hypothesis regarding the primary outcome, empowerment, was not supported. An increase in the secondary outcome, satisfaction, was observed, although the effect size was small, and results should be interpreted with caution. Findings from this staff educational intervention can inform the development of future studies aimed at improvement of inpatient care for persons with severe mental illness.

**Trial registration:**

The trial was retrospectively registered at ClinicalTrials.gov June 9, 2017, identifier: NCT03182283

## Background

Persons with psychotic disorders both can and want to participate in their own care [1–3]. Their views of appropriate goals and interventions often differ from those of formal care providers [4, 5], and patients’ concerns for their social needs (relationships, daily activities) are on par with those of their clinical needs [6]. However, patients experience that opportunities for involvement are lacking [7], which can contribute to dissatisfaction with care [8]. While potential advantages of the involvement of persons with complex and chronic conditions in their own care were recognized by the WHO over a decade ago [9], and emphasized more recently [10], patient participation in mental health care seems to exist primarily at a policy level [11]. According to the authors of a recent review [12], reluctance on the part of mental health professionals constitutes an important barrier to patient participation. Clinical strategies that are acceptable to both patients and staff need to be developed and implemented.

One such an approach, which brings the patient’s participation to the forefront is Person-Centered Care (PCC). Although definitions of PCC vary, a common basic assumption is that health care staff must recognize the patient as a *capable* person, with own experiences, knowledge, and preferences. Focus is shifted from the *disease* to the *health* of the patient. The starting point is the patient’s own context and priorities, which means that staff must listen to the patient’s own narrative [13]. Acknowledging the patient’s own capacity makes it possible for staff and patients to work as partners in the creation of a personalized plan to improve health. Principles of PCC interventions highlighted in a recent concept review included empowerment, personhood, and individualized care [12]. Concept analyses and frameworks suggest that PCC is likely to improve quality and involvement in care, increase satisfaction with care, and improve health outcomes and well-being [14–16]. In somatic care settings, PCC interventions have been associated with increased patient satisfaction and quality of care [17], as well as increased self-efficacy, shorter hospital stay, and better functional performance [18–21]. Reduced agitation and increased quality of life have been shown for persons residing in old age care settings after PCC interventions [22–25]. In psychiatric care, however, outcomes of PCC are less often reported. A recent review on PCC in inpatient settings [16] identified only 3 original research papers reporting outcomes after a PCC intervention [26–28]. Two of these were set in a psychosis care setting. One [26] was a pilot study that tested an electronic care planning system. Decreased symptoms of aggressive behavior, depression, withdrawal and psychosis were observed post-intervention. The other study [27] involved an IT-based patient education service which facilitated a closer patient-nurse relationship, individualized support, and increased self-efficacy in patients. In diagnostically mixed psychiatric inpatient settings, PCC has been used as one successful feature in a complex intervention to reduce the use of restraints [28, 29]. A practice development paper described promising results after the introduction of a person-centered care plan; both staff and patients reported enhanced patient involvement in care [30].

While the above-cited studies suggest that components of PCC can seem promising for psychiatric inpatient settings, we could find no studies specifically focusing on educational interventions that target hospital staff to increase the level of person-centered care for persons with schizophrenia and similar psychoses. Therefore, we developed a hospital-based multi-professional educational intervention designed to impact on staff attitudes and routines to stimulate increased person-centeredness in the inpatient care of persons with schizophrenia and similar psychoses [31]. The purpose of the current study was to compare patient-related outcomes before and after implementation of the educational intervention. We hypothesized that we would observe increased patient empowerment and increased patient satisfaction with care after implementation of the intervention.

## Methods

### Study design

The present study is the first of several planned studies describing outcomes from the project Person-Centered Psychosis Care (PCPC) – a staff educational intervention and implementation [31]; clinicaltrials.gov, identifier: NCT03182283. In the current study, a before and after design was used to assess patient outcomes associated with the PCPC intervention. The reporting of this study follows the CONSORT guidelines for non-pharmacologic randomized trials [32] on all points applicable to a before and after design.

### Study setting

The study took place at the Psychosis Clinic, Sahlgrenska University Hospital, which provides all inpatient care for patients with psychotic disorders in Gothenburg, Sweden’s second largest city. The inpatient services include four wards with a total of 43 beds, and all wards participated in the intervention.

### The PCPC intervention

PCPC is an intervention that aims to develop a more person-centered care on inpatient wards for persons with schizophrenia spectrum disorders. We followed the framework established by Gothenburg Center for Person-Centred Care (GPCC) to operationalize the PCPC intervention. The GPCC framework focuses on capturing the patients narrative (initiating a partnership), creating care plans with the patient as an active partner (working the partnership), and documenting agreements (safeguarding the partnership) [13].

**Fig. 1:**
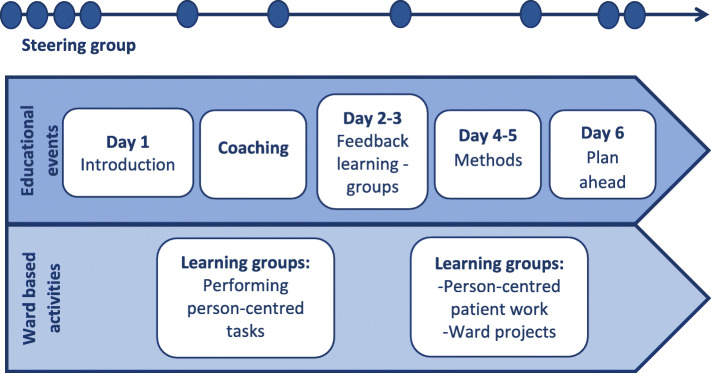
The educational and experimental learning phase process. Figure 1 was originally published in our study protocol, Goulding et al. (2018), BMC Psychiatry by Springer Nature and is used here under the Creative Common License (http://creativecommons.org/licenses/by/4.0/)

PCPC was designed with two overlapping phases. The first was an educational intervention that used a participatory design [33]. Staff members took part in lectures, workshops, and experimental learning. Lectures provided a theoretical knowledge base on person-centered care and implementation processes. Invited guests from outpatient services as well as patients with ongoing service contact participated and contributed with their knowledge and perspectives. Experimental learning involved practicing the features of the GPCC framework and creating own projects to increase person-centeredness in everyday care on the wards. The educational phase consisted of 6 days of coursework spread over a 6-month period, interspersed with practical ward-based projects testing features of PCC (see Fig. 1). The latter were supported by supervision and coaching by an outside facilitator. One third of all staff (across all professions and roles, including psychiatrists) at all four hospital wards at the Psychosis Clinic participated. In order to involve all staff in the intervention process, course participants exchanged experiences, ideas, and reflections with the rest of staff in knowledge translation activities designed to increase knowledge and awareness also in staff members who did not take part in the actual coursework. Knowledge translation activities included ward meetings for all staff, group sessions for staff supervised by PCC experts, as well as lunch dates. The latter were working lunches during which a staff member who had taken the PCC course met with two colleagues who had not, in order to facilitate knowledge exchange.

The second phase involved the implementation of staff-initiated projects and new ward routines. Examples included creating a structure to allow time and place to listen to the patient’s narrative, increasing patient participation in the creation of the care plan (writing the plan together with patients or using the patient’s own words), improving written information to patients, altering round routines to include patient participation, and improving cooperation with outpatient clinics to facilitate person-centered transitions from inpatient to outpatient care. As described in our study protocol [31], a steering group and an implementation group, with members from each ward, monitored the development of person-centered care activities and provided support when needed during the implementation period. Lectures and seminars were held, and a senior psychiatrist along with several of the clinic’s specialists in psychiatric nursing supervised small group sessions where various aspects of person-centered care were discussed. These procedures facilitated change from within by creating an environment that encouraged continuous improvement of care without reliance on external resources.

### Participants

All persons receiving inpatient care at any of the clinic’s four wards during the measuring periods were potentially eligible if meeting the following criteria: age ≥ 18 years and fulfilling criteria for a clinical diagnosis in the schizophrenia spectrum disorder (F20-F29) in accordance with ICD-10 [34]. Exclusion criteria were severe cognitive disability with inability to comprehend study goals and procedures (as determined by the patient’s psychiatrist), or lack of knowledge of the Swedish language to such a degree that an interpreter was required. The aim was to include 50 patients in each group (pre- and post-intervention) and sampling continued until each group was filled.

### Measures

The primary outcome measure was patients’ self-reported empowerment, which is an important feature of person-centered care. The *Empowerment Scale* [35, 36] was used, which consists of 28 items with responses given on a four point Likert-type scale ranging from “totally agree” to “totally disagree”. Positive and negative statements are mixed and some items are reversed when calculating the total score so that a higher score represents greater perceived empowerment. The mean item score (the total score divided by number of items) is used to illustrate the level of empowerment. Items correspond to 5 factors of empowerment (Self-esteem – self-efficacy: i.e. “I see myself as a capable person”, Power – powerlessness: “You can’t fight city hall”, Community activism and autonomy: “People have the right to make their own decisions even if they are bad ones”, Optimism and control over the future: “Very often a problem can be solved by taking action”, and Righteous anger: “Making waves never gets you anywhere”). The scale has shown good internal consistency (Cronbach’s alpha = .86) and the five factors explained 54% of the variance in a validity study [35]. The scale is validated for and used internationally in studies involving persons with severe mental illness [36]. Cronbach’s alpha in the current sample was .75.

The secondary outcome measure was self-reported consumer satisfaction with health care, measured with the *UKU-ConSat Rating Scale* [37]. The scale contains 11 items; responses are given on a seven-point scale ranging from − 3 (very bad/negative/little) to + 3 (very good/positive/much) reflecting levels of satisfaction with different aspects of the care provided (e.g. opportunity to get information on care decisions, participate in decision-making, medication, and psychosocial interventions). All total scores above zero are considered to reflect satisfaction and the level of satisfaction increases with the score. Correspondingly, scores below zero reflect different levels of dissatisfaction. The ConSat scale is constructed and validated for psychiatric patients, including both in- and outpatients with psychotic disorders [37, 38]. This study used the self-rating version for consumers which has good internal consistency (Cronbach’s alpha = .80). Correlations with the original interview version of the scale showed Pearson *r* ranging from .67 to .82 [39]. Cronbach’s alpha in our PCPC sample was .88.

Possible confounders including symptom burden, functional ability, and overall health were quantified for each patient at study inclusion. Symptom burden was rated with the *Remission sub-scale* (RSS) of the *Positive and Negative Syndrome Scale*, which consists of 8 items that reflect core symptoms of schizophrenia [40]. The scores (1 = non-existing symptom to 7 = extremely severe symptom) were based on interviews with the patient and his/her contact person (staff). Functional ability was determined using the *GAF Scale* [41] which grades function on a 1–100 scale (100 = fully functional in all life domains). Scoring was done by the participant’s contact person at the hospital ward. Overall health was self-rated with the *EQ-5D Scale* [42], which consists of 5 items that reflect function and discomforts and yields a total health index ranging from - 0,594 to 1 (full health). The EQ-5D scale also includes a visual analogue scale rating subjective level of overall health. The overall health scale ranges between 1 and 100 (best imaginable health).

### Procedure

Patients were recruited for the pre-intervention group from May 2014 until December 2014. The educational intervention took place from December 2014 until May 2015, followed by implementation work. Post-intervention recruitment took place from May 2017 until February 2018. Patients who were soon to be discharged and met the inclusion criteria were informed about the study by one of the authors (KA, AG or LA) if available on days when the researchers were present at the clinic. All participants were given written and oral information about the study goals, procedures, and data management. They were informed that participation was voluntary and that it was possible to withdraw from participation without having to give any explanation. All signed a consent form before participating in any study-related task, and all received a copy of the signed consent and study information. The procedures conformed with the principles outlined in the Declaration of Helsinki [43] and were approved by the Regional Ethical Review Board (DNr 773–13). A researcher administered the self-rating questionnaires, assisting patients when needed. All data collection took place on the wards prior to discharge. Core symptoms were rated by the researcher with the RSS. The staff member assigned as the patient’s contact person was interviewed to complete the observer section of the RSS and the GAF assessment. Information regarding age, diagnosis, form of care (voluntary/compulsory), and length of inpatient stay was collected from medical records.

### Statistical analyses

The patient sample size was based on a power calculation of the primary outcome (Empowerment). The power calculation was based on independent samples t-test and showed that a total sample size of 84 participants (42 per group in a balanced design) yields 80% power to detect a .2 difference in mean item score between groups when pre-intervention and post-intervention scores are compared with a two-tailed test at a significance level of .05.

The distributions of the continuous variables were studied to determine whether parametric or non-parametric analyses could be employed when analyzing group differences. The Chi-square test was used to analyze dichotomized data and Students t-test for continuous variables in background and possible covariate data. Two-tailed tests were used and *p*-values less than .05 were considered statistically significant.

Data distributions allowed parametric testing for both the Empowerment and UKU ConSat scales. ANCOVAs were used to analyze group differences, adjusting for covariates. Four potential outliers were identified among the pre-implementation participants regarding Empowerment. Two of these persons rated high levels of empowerment, two rated low levels. Four potential outliers were identified in the UKU ConSat data, one among the pre- and three among the post-intervention participants. These persons rated low levels of satisfaction. Analyses were carried out both with and without the outlier cases.

Ratings with single missing items were included in the analyses and handled as follows. A single missing item on the Empowerment scale was replaced by the participant’s subscale mean. A single missing item on the UKU-ConSat was replaced by the middle alternative (0). In accordance with EQ-5D instructions [44], missing items were left blank when calculating the index score. One participant could not complete the empowerment scale due to fatigue; the rating was thus excluded. Two further participants were too fatigued to complete the RSS interview. GAF data were missing for one of these as well as for another participant, due to staff unavailability.

## Results

Patients fulfilling inclusion criteria (*n* = 185) were approached, and 102 agreed to participate. Two of these started to fill out the questionnaires but chose not to continue after a few questions and were therefore excluded. The 83 approached patients who declined participation (labelled non-participants), did so for two main reasons; they did not want to participate (pre-implementation non-participants *n* = 43; post-implementation non-participants *n* = 34) or they wanted to participate but did not want to sign the consent form or wanted a monetary compensation for participating (pre-implementation non-participants *n* = 5; post-implementation non-participants *n* = 1).

Table 1 shows patient characteristics by participations status. Almost half of the participants were women. Most were admitted in accordance with involuntary care legislation. Schizophrenia and Unspecified non-organic psychosis were the most common diagnoses. The mean length of stay was 50.7 days (*SD* = 49.1, *Md* = 39.0) for the pre-implementation participants and 64.6 days (*SD* = 55.5, *Md* = 49.5) for the post-implementation participants. There were no differences between groups regarding age, gender, diagnosis or involuntary care status.

**Table 1:** Patient characteristics by participation status

Characteristic	Pre-implementation		Post-implementation	
	Participants (*n* = 50)	Non-participants (*n* = 48)	Participants (*n* = 50)	Non-participants (*n* = 35)
Mean age (*SD*)	48.0 (14.7)	49.9 (13.9)	46.9 (15.4)	46.4 (14.1)
Age range	20–78	27–77	19–84	21–88
	*n (%)*	*n (%)*	*n (%)*	*n (%)*
Women	23 (46)	24 (50)	20 (40)	15 (43)
Involuntary care	35 (70)	39 (81)	31 (62)	25 (71)
Schizophrenia	16 (32)	26 (54)	15 (30)	13 (37)
Schizoaffective disorder	11 (22)	13 (27)	10 (20)	7 (20)
Delusional disorder	6 (12)	2 (4)	10 (20)	3 (9)
Unspecified nonorganic psychosis	17 (34)	7 (15)	15 (30)	12 (34)

Fisher’s exact test, t-test, Chi^2^-test, and Mann-Whitney U-test were used to test differences between participants and non-participants. In the pre-implementation sample, the proportion of persons with a clinical diagnosis of schizophrenia was significantly greater among non-participants compared with participants who more often had a non-specified psychosis diagnosis (Fisher’s exact test = 8.52, two-tailed *p* = .035). No significant differences were detected between the two groups regarding age, gender, length of stay or proportions with involuntary care. There were no significant differences between participants and non-participants in the post-implementation sample.

Of the three variables included as possible confounders, both overall health, as measured by EQ-5D, and function, as measured by GAF, were significantly lower in the post-implementation group, see Table 2. No such difference was detected regarding the rating of core psychotic symptoms (RSS). As EQ-5D and GAF correlated significantly (Pearson *r* = .31, *p* = .002), we decided to use EQ-5D as a covariate in the ANCOVA analyses since this measure was considered more reliable and valid. A post hoc analysis of the ConSat scale item 8, which rates satisfaction with medication, was conducted to explore potential differences related to medication satisfaction. No difference was found between the pre- and post-intervention groups (*M* = 0.32 vs 0.43; *t*(98) = −.292, *p* = .771).

**Table 2:** Ratings of background and outcome variables in patient participants before and after PCPC implementation

	Pre-implementation participants (*n* = 50)^a^	Post-implementation participants (*n* = 50)	Two-tailed *t (p)*	95% CI of mean difference
Measure	*M (SD)*	*M (SD)*		
**Background variables**				
EQ-5D Index	.62 (.38)	-.05 (.31)	9.56 (<.0005)	.53–.81
EQ-5D VAS	65.99 (25.04)	52.76 (24.47)	2.67 (.009)	3.40–23.06
GAF Function subscale	67.06 (15.12)	55.22 (13.60)	4.08 (<.0005)	6.08–17.60
GAF Symptom subscale	60.24 (15.78)	48.86 (15.13)	3.67 (<.0005)	5.22–17.55
PANSS RSS	14.67 (5.56)	13.10 (4.19)	1.58 (.117)	-.40–3.53
**Outcome variables**				
Empowerment Scale	2.95 (.29)	2.91 (.29)		
UKU ConSat Scale	7.11 (12.44)	9.06 (13.15)		

^a^Due to missing data in the pre-intervention sample, GAF Function subscale has *n* = 48, GAF Symptom subscale *n* = 49, PANSS RSS *n* = 48 and Empowerment Scale *n* = 49

Table 2 shows mean Empowerment and Consumer Satisfaction scores for the pre- and post-intervention groups. Empowerment item mean scores ranged from 2.29–3.89 in the pre-implementation group, and 2.21–3.57 post-intervention. Corresponding figures for satisfaction total scores were  -33–27 and  -26–31 respectively.

The estimated marginal mean Empowerment value was numerically higher in the post-intervention group but the difference was not significant, and excluding outliers reduced the difference further (Table 3). Thus, the hypothesis regarding the primary outcome, increased empowerment, was not supported.

**Table 3:** Estimated marginal means and group difference results regarding Empowerment and Consumer satisfaction

Measure	Pre-implementation participants	Post-implementation participants	95% CI of mean difference	*F (p)*
	Estimated marginal mean^a^ (*SE*)	Estimated marginal mean^a^ (*SE*)		
**Empowerment**				
With outliers	2.87 (.048)	2.99 (.047)	-.27–.04	2.2 (.142) *eta*^*2*^ = .02
	*n* = 49	*n* = 50		
Without outliers	2.90 (.047)	2.96 (.043)	-.21–.09	.64 (.426) *eta*^*2*^ = .007
	*n* = 45	*n* = 50		
**Consumer satisfaction**				
With outliers	4.46 (2.15)	11.71 (2.15)	-14.17– -.31	4.29 (.041) *eta*^*2*^ = .04
	*n* = 50	*n* = 50		
Without outliers	5.81 (1.78)	13.46 (1.89)	-13.44– -1.87	6.91 (.010) *eta*^*2*^ = .06
	*n* = 49	*n* = 47		

^a^EQ-5D index mean was used as covariate in the ANCOVA of both Empowerment and Consumer satisfaction data

The estimated marginal mean for Consumer satisfaction was significantly higher in the post-intervention group compared to the pre-intervention group (Table 3). The hypothesis regarding the secondary outcome, increased satisfaction, was thus supported. The difference between the groups was more pronounced after the exclusion of outliers (Table 3).

## Discussion

We applied a participatory approach to the development and implementation of a multi-professional educational intervention for staff involved in inpatient care for persons with schizophrenia spectrum disorders. We did not find support for our hypothesis that empowerment would be significantly higher in the post-intervention group, but an increase in patient satisfaction with care was observed, although the effect size was small. As publications on participatory approaches to increase PCC are rare, especially in the psychiatric setting, our results can help to inform the development and testing of new approaches to inpatient care for persons with schizophrenia and similar psychoses.

There may be different explanations for the observed lack of improvement in empowerment. First, it is possible that our intervention did not actually improve the level of person-centeredness on the wards to a degree sufficient to impact on patients’ feelings of empowerment. Another possibility is that the intervention did indeed improve person-centeredness, but that the latter did not affect empowerment. This is however theoretically contradictory since empowerment is tightly connected with several aspects of PCC and stressed in PCC concept analyses [12, 45]. The phenomenon of empowerment encompasses numerous elements related to the experience of being in control of determinants of quality of life, including health, relationships, work/economy, and living/home [46]. While the PCPC intervention might affect several of these aspects, it targets mainly health-related issues, and it is possible that affecting this particular aspect does not raise the overall experience of empowerment. Further, the empowerment scale employed in this study captures empowerment on an overall level. The measure was constructed on the basis of mental health service users’ own perspectives of empowerment, which is in line with PCC. However, the scale addresses the whole life perspective and some items (for example “You can’t fight city hall”, “People are limited only by what they think possible”) might lack relevance in our clinical context. It is possible that a scale focusing more specifically on empowerment in care situations would yield a different result.

We did find support for our hypothesis that the secondary outcome, patient satisfaction, would be greater in the post-intervention group. However, this result should be interpreted with caution as patient satisfaction was our secondary outcome and the effect size was small. The finding expands on previous results from person-centered interventions in other settings including outpatient services for persons with psychotic illness [47] and inpatient somatic care [17]. The PCPC intervention focused highly on staff-patient relations, and good relations with staff have been linked to patient satisfaction in inpatient psychiatric care in a recent review [48]. It is possible that the intervention contributed to an improvement in overall ward atmosphere, which has also been shown to contribute to inpatient satisfaction [49]. Interventions designed to improve patients’ own experiences of care may have implications for prognosis, as patient satisfaction has been linked to future treatment outcomes [50].

The pre-intervention patient satisfaction score was almost identical to that reported from a recent Danish inpatient study investigating satisfaction with care using the same instrument [51], and indicates that patients in both studies were somewhat satisfied in view of the interpretation that all scores above 0 denote satisfaction. Improvement in patient satisfaction in the post-intervention group was observed despite the large proportion of patients with involuntary admission. Involuntary treatment has been shown to have a negative impact on satisfaction [48, 52] although it has been argued that person-centered care can co-exist with involuntary treatment [53]. In a qualitative study of service users’ experiences during involuntary care, positive and empowering experiences were described in the narratives [54]. The authors of the latter study concluded that informative and collaborative approaches to care may reduce the traumatic impact of involuntary admissions.

When planning this study, we were aware of possible confounding variables that might affect the results [31]. Therefore, we planned to conduct ANCOVA:s if data permitted parametric analyses. Overall health turned out to be a confounder and was therefore included as a covariate in the analyses of the outcome variables. Other possible confounding variables are age, legal status (voluntary vs involuntary care), and severity of symptoms, all of which have been linked to satisfaction in previous research [55]. This study did not find any differences regarding age, legal status or symptom severity between the pre- and post-intervention groups, therefore these variables were not suspected to be confounders here. Another possible confounding factor when studying patient satisfaction is pharmacological treatment. If participants experience negative side effects or limited medication effects, this might decrease their satisfaction with care [8, 56]. As satisfaction with medication is one of the items rated in the UKU ConSat Scale, we carried out an explorative analysis of this single item. Similar ratings were observed in the pre- and post-intervention groups, suggesting that satisfaction with pharmacological treatment was not a confounder here. However, it must be stressed that a scale designed specifically for the rating of medication side effects and efficacy might have identified confounding factors that we were unable to address in the current study.

The intervention was carried out in a real-world context, and used a participatory approach to ensure that it was fitted to the everyday clinical context. The participatory approach is anticipated to facilitate sustainable change since it builds on involvement of staff throughout the development and implementation process [57]. Initially, we considered a manualized intervention involving a monitoring research nurse. However, after discussions with an expert on the design of interventions to promote change in complex organizations, we opted for the participatory approach to boost both sustainability and ecological validity. Such an approach has successfully been used to implement PCC in other Swedish settings [58]. A major limitation of this approach is reduced reproducibility as the intervention is tailored for specific contexts. However, the intervention does come with a degree of standardization since it is based on core features as described in the Gothenburg framework for Person-Centered Care [13]. Adopting a participatory approach means that staff members are involved in *how* these core features should be implemented in their specific care context.

This study evaluates a complex intervention, which entails difficulties. It is not easy to determine the level of implementation, or “dose” of the intervention, as PCC is not a method for care provision, but rather an approach to care. The intervention was meant to shape staff attitudes and routines. While there are now several tools measuring aspects or proxies of PCC in different contexts [59, 60], none had been applied and validated in the context of care for persons with psychotic illness when we designed the current study. Reports provided by staff representatives at steering group meetings and audits of person-centered care plans were used to determine whether PCPC activities had been absorbed into everyday ward practice before conducting the post-intervention data collection. Although these reports and observations suggested an uptake of PCC, we still do not know the extent to which everyday care situations were actually person-centered. Previous studies show that the degree of engagement and interest in PCC implementation varies among staff [61], as do their perceptions of what actually constitutes PCC [62]. This might contribute to a high level of heterogeneity in the care delivered. It has also been shown that even among staff who perceive their care to be person-centered, many care situations remain “traditional” i.e. more paternalistic [63]. As health professionals may overestimate their own person-centeredness [63, 64], the addition of an objective measure of person-centeredness would constitute an important addition in future studies to determine actual implementation of person-centered practices, along with a process analysis to increase the understanding of results [65].

Another factor, related to the real world context, is staff turnover which has previously been found to negatively impact the engagement in PCC implementation [62]. During the PCPC implementation there was considerable turnover of both front-line staff and management. This meant that the time between the educational intervention and follow-up had to be prolonged in order to allow more time for implementation of staff PCPC projects into everyday ward routines.

There are other limitations that need to be discussed. The relatively small number of participants in our study made it necessary to limit the number of outcome variables. Our study lacked a recovery outcome, which is stressed by people using mental health services and researchers alike [66, 67]. One reason for not including such an outcome was the relatively short time period for inpatient care. The recovery process is expected to continue long after discharge, and thus be influenced by attitudes and routines in outpatient services. While all outpatient care providers in the catchment area utilize the Resource group - Assertive Community Treatment (R-ACT) approach [47], the degree to which this is applied varies across services.

The before and after design is a major limitation since it prevents us from drawing conclusions regarding which factors might have contributed to the observed change in consumer satisfaction. Even though randomized controlled trial protocols (RCT) have been “translated” to suit complex health care interventions [68], it is not always possible or even optimal to use an RCT design when assessing interventions in such a context [69]. Randomizing individual patients in this study would risk contamination since the intervention introduces a new way of thinking. Staff cannot be expected to “turn off” the PCPC care approach for control group patients. Randomizing two of the four wards to the PCPC intervention and two to treatment as usual was not an option since hospital logistics sometimes require that staff and patients be moved between wards. For the same reason a cross-over design was not possible. A larger RCT design with randomization at group level (service providers randomized) would be more optimal but that would be an expensive endeavor, taking the project to a regional or national level.

A randomization sampling procedure was not possible in this study. Sampling bias was decreased by approaching all eligible patients who were available on data collection days. The enrollment rate must be considered a limitation as almost half of those asked to participate declined. However, the patient characteristic data presented in Table 1 shows that participants and non-participants were similar regarding almost all measured variables, indicating that our participants can be considered representative for this psychosis care setting. We cannot generalize our findings to patients with severe cognitive impairment, severe psychotic symptoms or those who lack Swedish language competence, as these were not able to participate in the study. Another type of study design would be needed to capture these patient groups. An observational study could be an alternative for those with severe impairment. Translator services would facilitate participation of those who do not speak Swedish.

## Conclusions

The current study did not find the hypothesized increase of patient empowerment following a participatory staff intervention aiming to increase PCC in inpatient psychosis units. The results indicate that patient care satisfaction increased. This is however a secondary outcome with low effect size and is to be interpreted with caution. This paper can help to inform the future development of hospital-based PCC for persons with schizophrenia and similar psychoses. Coming papers from this project will report outcomes on ward level data as well as staff, patient and next-of-kin experiences, which will contribute to the overall understanding of PCC in a psychiatric setting.

## Data Availability

In accordance with our ethics review board, the datasets generated and analyzed during the current study are not publicly available for ethical reasons, but are available from the corresponding author on reasonable request.

## Abbreviations

ANCOVA: Analysis of Covariance
CI: Confidence Interval
GAF: Global Assessment of Functioning
GPCC: Gothenburg Center for Person-Centred Care
ICD-10: International Statistical Classification of Diseases and Related Health Problems - Tenth Revision
PANSS: Positive and Negative Syndrome Scale
PCC: Person-Centered Care
PCPC: Person-Centered Psychosis Care
RCT: Randomized Controlled Trial
SD: Standard Deviation
UKU-ConSat: UKU-Consumer Satisfaction Scale
WHO: World Health Organization
